# A New Dental Journal

**Published:** 1890-08

**Authors:** 


A NEW DENTAL JOURNAL, under the title of *The Dental Mirror*, Dr. R. Ottolengui as editor, issued the first number in June last. Dr. Ottolengui is well known as a contributor to dental literature, and no doubt but that his new journal will meet with great success.

